# Wild Raspberry Subjected to Simulated Gastrointestinal Digestion Improves the Protective Capacity against Ethyl Carbamate-Induced Oxidative Damage in Caco-2 Cells

**DOI:** 10.1155/2016/3297363

**Published:** 2015-12-16

**Authors:** Wei Chen, Yang Xu, Lingxia Zhang, Ya Li, Xiaodong Zheng

**Affiliations:** ^1^Department of Food Science and Nutrition, Zhejiang Key Laboratory for Agro-Food Processing, Zhejiang University, Hangzhou 310058, China; ^2^College of Food Science and Biotechnology, Zhejiang Gongshang University, Hangzhou 310035, China

## Abstract

Ethyl carbamate (EC), a probable human carcinogen, occurs widely in many fermented foods. Previous studies indicated that EC-induced cytotoxicity was associated with oxidative stress. Wild raspberries are rich in polyphenolic compounds, which possess potent antioxidant activity. This study was conducted to investigate the protective effect of wild raspberry extracts produced before (RE) and after *in vitro* simulated gastrointestinal digestion (RD) on EC-induced oxidative damage in Caco-2 cells. Our primary data showed that ethyl carbamate could result in cytotoxicity and genotoxicity in Caco-2 cells and raspberry extract after digestion (RD) may be more effective than that before digestion (RE) in attenuating toxicity caused by ethyl carbamate. Further investigation by fluorescence microscope revealed that RD may significantly ameliorate EC-induced oxidative damage by scavenging the overproduction of intracellular reactive oxygen species (ROS), maintaining mitochondrial function and preventing glutathione (GSH) depletion. In addition, HPLC-ESI-MS results showed that the contents of identified polyphenolic compounds (esculin, kaempferol O-hexoside, and pelargonidin O-hexoside) were remarkably increased after digestion, which might be related to the better protective effect of RD. Overall, our results demonstrated that raspberry extract undergoing simulated gastrointestinal digestion may improve the protective effect against EC-induced oxidative damage in Caco-2 cells.

## 1. Introduction

Ethyl carbamate (EC) was initially identified as a carcinogen to animals in the 1940s and subsequently classified as a group 2A carcinogen that is probably carcinogenic to humans by IARC, a World Health Organization's International Agency for Research on Cancer [1]. It was noticeable that ethyl carbamate was detected in many fermented foods, particularly in yeast breads and alcoholic beverages [2]. Generally, the formation of EC in those fermented foods was based on the reaction between ethanol and nitrogen-containing compounds such as urea and citrulline [3]. Fermented foods, due to its good taste, have become an important part of human diets. However, recent studies carried out in mice have revealed that frequent exposure to EC may cause genotoxicity and cytotoxicity and even leads to cancer development [4–6]. Therefore, potential harm of EC on human health cannot be neglected.

Recently, accumulating evidence unveils that EC-induced toxicity is associated with cellular oxidative stress. For example, Chun et al. suggested that the excessive production of intracellular ROS induced by EC may create a persistent oxidative stress environment promoting lung epithelial cells transformation, thereby resulting in cancer development [7]. Polyphenols exist widely in foodstuffs including vegetables and fruits [8] and are capable of preventing oxidative damage-related diseases [9]. Therefore, increasing attentions have been paid to explore naturally occurring antioxidants derived from vegetables and fruits for protection against EC-induced toxicity.

Raspberry is well known to contain massive phytochemicals such as flavones, flavonols, anthocyanins, and phenolic acids [10], which could ameliorate cellular oxidative stress [9]. It is noticeable that foodstuff will pass through gastrointestinal tract and be exposed to gastrointestinal condition before providing specific health benefits. As a consequence, some functional components may be transformed into other compounds with varied bioactivity [11]. Although aforementioned studies have addressed that raspberry extract possessed potent antioxidant activity, the simulated gastrointestinal condition should be taken into consideration to evaluate the real effect of raspberry extract* in vitro*. Therefore, our study aims to elucidate the protective effect of raspberry subjected to* in vitro* simulated gastrointestinal digestion on EC-induced oxidative stress in Caco-2 cells.

## 2. Materials and Methods

### 2.1. Materials and Reagents

Wild raspberry fruits (*Rubus hirsutus *Thunb.) were gathered from Siming Mountain in Zhejiang Province, China. Fresh fruits were washed with distilled water and the water absorbed on the surface of fruits was then dried in dust free environment at room temperature. After that, the fruits were screened through maturity, size, and integrity and then stored at −80°C in a refrigerator prior to use. 3-(4,5-Dimethyl-2-thiazolyl)-2,5-diphenyl-2-H-tetrazolium bromide (MTT), dihydroethidium (DHE), 2′,7′-dichlorofluorescin diacetate (DCFH-DA), Nonyl Acridine Orange (NAO), Rhodamine 123 (Rh123), Hoechst 33258, Folin & Ciocalteu's phenol reagent, pepsin, pancreatin, and bile salts were purchased from Sigma-Aldrich (St. Louis, MO, USA). Total superoxide dismutase assay kit and cell lysis buffer were purchased from Beyotime Institute of Biotechnology, Ltd. (Shanghai, China). All other reagents used were of analytical grade.

### 2.2. *In Vitro* Digestion

The* in vitro* digestion method previously described by Faller et al. [12] was modified and then used for simulating gastrointestinal condition in this work. Briefly, 20 g of wild raspberry fruit was homogenized and diluted to a volume of 20 mL with distilled water. For the gastric digestion stage, 5 M HCL was added to adjust pH value to 2, with the porcine pepsin (6000 units) added subsequently. The mixture was then incubated at 37°C in a shaking water bath for 1.5 h at 100 rpm. For the intestinal digestion stage, the pH of mixture undergoing gastric digestion was adjusted to 6.5 with 1 M sodium bicarbonate and then 5 mL of pancreatin was added (consisting of 25 mg/mL porcine bile salts and 4 mg/mL trypsin). Subsequently, pH was immediately adjusted to 7.4 using 1 M sodium hydroxide. Thereafter, the mixture was incubated at 37°C in a shaking water bath for 2 h at 100 rpm. At the end of incubation, the mixture was diluted to a volume of 25 mL with distilled water and then centrifuged at 5000 rpm for 6 min. Supernatant was collected, named as raspberry digesta (RD). The wild raspberry fruit without* in vitro *digestion was named as raspberry extract (RE). Both RE and RD were stored in −80°C for further investigation.

### 2.3. Chemical Characterization

#### 2.3.1. Total Phenolics Determination

Total phenolic content was determined using Folin-Ciocalteau method. Briefly, 0.1 mL RE or RD solution diluted with 0.5 mL distilled water was mixed with 0.1 mL Folin-Ciocalteau reagent and then shaked in a water bath for 5 min. After that, 0.2 mL sodium carbonate was added to the mixture and then diluted to a final volume of 1 mL with distilled water before incubating at room temperature for 2 h. The absorbance of the mixture was measured at 760 nm. Gallic acid was used as a standard and results were expressed as mg of gallic acid equivalents (GAE) per 100 g fresh weight.

#### 2.3.2. Total Flavonoids Determination

The colorimetric assay described by Zhishen et al. [13] was used to determine the total flavonoids content. Briefly, 0.04 mL sodium carbonate (5%, m/v), 0.5 mL RD solution, and 0.5 mL distilled water were mixed and then incubated for 5 min. After that, 0.04 mL aluminium nitrate (10%, m/v) was added and diluted to a final volume of 1 mL with distilled water. The mixture was incubated at room temperature for 15 min and its absorbance was measured at 510 nm. Rutin was used as a standard and the total flavonoids content was expressed as mg of rutin equivalents (RE) per 100 g fresh weight.

#### 2.3.3. Phenolic Compounds Identification by HPLC-ESI-MS

Before analyzing by Waters UPLC system equipped with Promosil C18 column (4.6 × 250 mm, 5 *μ*m) and a Triple-TOF Mass Spectrometry System (AB SCIEX, Triple-TOF 5600 plus Framingham, USA), wild raspberry extracts produced before and after* in vitro* digestion were filtered through 0.45 *μ*m membrane. The eluent consisted of 0.1% formic acid aqueous solution (A) and methanol (B), and the elution process was performed by the following linear gradient: from 95% to 85.8% A for 20 min, from 85.8% to 40% A for 50 min, from 40% to 95% for 10 min, and then isocratic elution for 5 min. with the inject volume of 10 *μ*L and flow rate of 0.8 mL/min. The MS conditions were listed as follows: detection was performed in negative ion modes at a temperature of 550°C and voltage of 4.5 KV, the scan range (*m/z*) ranged from 100 to 2000, and the UV detector set at 260 nm. Identification was based on the ion molecular mass, MS^2^ and UV-visible spectra data. Through the peak area, the content of identified compounds in raspberry extracts was compared.

### 2.4. Cell Culture

Human Caco-2 cells were obtained from the Cell Bank of Type Culture Collection of Chinese Academy of Sciences. Caco-2 cells were cultured in RPMI 1640 medium (Gibco) containing 10% of the new calf serum, 100 units/mL penicillin, and 100 units/mL streptomycin and incubated in a humidified incubator with 5% CO_2_ at 37°C.

For the control group, Caco-2 cells were incubated in the absence of EC, RE, and RD. For the EC group, Caco-2 cells were incubated only with EC for 24 h. For the RE group, Caco-2 cells were pretreated with RE for 2 h and then incubated with EC for 24 h. For the RD group, Caco-2 cells were pretreated with RD for 2 h and then incubated with EC for 24 h.

### 2.5. Cell Viability Assay

Cell viability was measured by the MTT method as previously described [14]. Briefly, the cells were seeded into 96-well cell culture plates, with the concentration of 5 × 10^3^ cells/well. Cells, which were pretreated with RE or RD for 2 h after incubation in cell culture plate for 24 h, were then cultured with ethyl carbamate (62.5 mM) for another 24 h. Subsequently, cells were incubated with MTT (0.5 mg/mL) for 4 h, and the generated formazan precipitate was dissolved with 150 *μ*L of DMSO. Finally, the absorbance was measured at 490 nm using a Tecan infinite M200 microplate reader.

### 2.6. Determination of Intracellular Reactive Oxygen Species

#### 2.6.1. DCF Fluorescence Assay

The fluorescent reaction that transmit nonfluorescent 2′,7′-dichlorofluorescein diacetate (H_2_DCFDA, Invitrogen) to fluorescent 2′,7′-dichlorofluorescein (DCF) was used to detect the intracellular reactive oxygen species [15]. Caco-2 cells were seeded into 12-well cell culture plates at a concentration of 1 × 10^5^ cells/well and cultured for 24 h. After that, cells were pretreated with RD (2 mg/mL) or RE (2 mg/mL) for 2 h and then incubated with ethyl carbamate (62.5 mM) for 24 h. Subsequently, cells were washed with PBS, collected and incubated with 10 *μ*M DCFH-DA at 37°C for 30 min, and then washed with PBS again and immediately determined by fluorescence microscope. The results were expressed as mean DCF fluorescence intensity calculated by image analysis software ImageProPlus 6.0 (Media Cybernetics, Inc.) from six different microscopic fields.

#### 2.6.2. DHE Fluorescence Assay

Intracellular superoxide anion radicals (O_2_^·−^) were analyzed using DHE fluorescence assay [16]. In brief, Caco-2 cells were subjected to the same treatment procedure mentioned in Section 2.6.1. The collected cells were incubated with 10 *μ*M DHE at 37°C for 30 min and then washed with PBS and immediately analyzed by fluorescence microscope. The results were expressed as mean DHE fluorescence intensity calculated by image analysis software ImageProPlus 6.0 from six different microscopic fields.

### 2.7. Determination of Cellular Glutathione (GSH)

Cellular glutathione was determined based on a previously described method with slight modifications [16]. Briefly, Caco-2 cells were treated according to the process mentioned in Section 2.6.1. Then cells were collected and incubated with 50 *μ*M NDA at 37°C for 30 min. After incubation with the fluorescence probe, cells were washed with PBS and analyzed by fluorescence microscope. The results were expressed as mean NDA fluorescence intensity calculated by image analysis software ImageProPlus 6.0 from six different microscopic fields.

### 2.8. Detection of Mitochondrial Membrane Potential (MMP)

Mitochondrial membrane potential (MMP) was measured using the method described by Chen et al. with some slight modifications [14, 17]. Briefly, cells were treated as the same process mentioned in Section 2.6.1. The collected cells were incubated with 10 *μ*g/mL RH123 at 37°C for 30 min. Then the cells were washed with PBS and immediately measured using fluorescence microscope. The results were expressed as mean RH123 fluorescence intensity calculated by image analysis software ImageProPlus 6.0 from six different microscopic fields.

### 2.9. Detection of Mitochondrial Membrane Lipid Peroxidation

According to the method previously described with some slight modifications [18], mitochondrial membrane lipid peroxidation was detected. Briefly, after treatment, the collected cells were incubated with 10 *μ*M of NAO at 37°C for 30 min, washed with PBS, and then detected by fluorescence microscope. The results were expressed as mean NAO fluorescence intensity calculated by image analysis software ImageProPlus 6.0 from six different microscopic fields.

### 2.10. Detection of Cell Nucleus Stained with Hoechst 33258

Hoechst 33258, a DNA-bound fluorescence dye, was used to observe the cell nucleus morphology according to the method with some modification [19]. Briefly, after treatment, the collected cells were incubated with 10 *μ*M of Hoechst 33258 at 37°C for 30 min. Then cells were washed with PBS and analyzed by fluorescence microscope.

### 2.11. Statistical Analysis

All experiments were carried out at least three times. The results were expressed as mean ± standard deviations (SD) and analyzed by one-way ANOVA using SPSS (version 19.0).* p* < 0.05 was considered to be significant.

## 3. Results and Discussion

### 3.1. Effect of RD on EC-Induced Cytotoxicity and Genotoxicity in Caco-2 Cells

**Figure 1:**
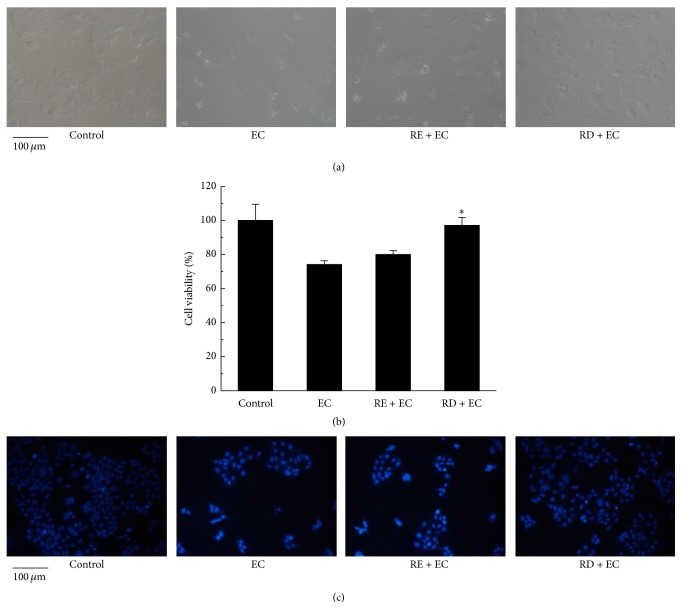
Effect of RD on EC-induced toxicity in Caco-2 cells. Caco-2 cells were incubated with 62.5 mM EC for 24 h in the presence or absence of RE (2 mg/mL) or RD (2 mg/mL). (a) Cell morphological image of Caco-2 cells. (b) The quantitative data of cell viability and results were expressed as mean percent (mean ± standard deviations). (c) Nuclear staining of Caco-2 cells with Hoechst 33258. ^*∗*^ *p* < 0.05 represents significant difference compared with EC group.

Cytotoxicity and genotoxicity induced by ethyl carbamate towards human cells have been reported previously [7, 20]. In this work, MTT assay and Caco-2 cells model were employed to observe the cytotoxicity induced by EC. As shown in Figures 1(a) and 1(b), after incubation with 62.5 mM EC for 24 h, the cell viability was remarkably decreased to 74.10%  ± 2.22% compared with that of control group (its cell viability is considered as 100%), which was in accordance with the results observed in RAW 264.7 cells by Chun et al. [7]. Therefore, Caco-2 cells model can be properly used to investigate the effect of RE or RD on EC-induced cytotoxicity. Before incubating with EC, Caco-2 cells were pretreated with RE (2 mg/mL) or RD (2 mg/mL) for 2 h. As we can see from Figure 1(b), the cell viability of RD pretreatment was increased by 23% compared with that of EC group, whereas that of RE pretreatment was only increased by 5.87%, which indicated that RD may effectively ameliorate EC-induced cytotoxicity. In addition, considering the genotoxicity generated by dietary EC exposure, the minor groove binder Hoechst 33258, which is known to sensitively bind the adenine-thymine rich sites of DNA, was used to detect the genotoxicity induced in Caco-2 cells and to evaluate the effect of RE or RD on EC-induced genotoxicity.  It could be observed from Figure 1(c) that most of nucleus in EC group contained small bright blue dots representing chromatin condensation or nuclear fragmentation compared with control group [21], whereas RD treatment group showed few bright blue dots, which demonstrated that RD pretreatment may exert a better performance in suppressing the toxicity to DNA induced by EC. Previous study unveiled that EC was metabolized to vinyl carbamate and then to vinyl carbamate epoxide* in vivo*; the latter can directly react with DNA, resulting in the DNA alkylation and the nucleic-acid adducts formation [22]. In our results, EC-induced DNA damage was ameliorated by treatment with RD. The possible explanation of this protective effect may be attributed to the reaction occurring between the metabolites and raspberry digesta.

### 3.2. RD Suppressed the Production of EC-Induced Reactive Oxygen Species

**Figure 2:**
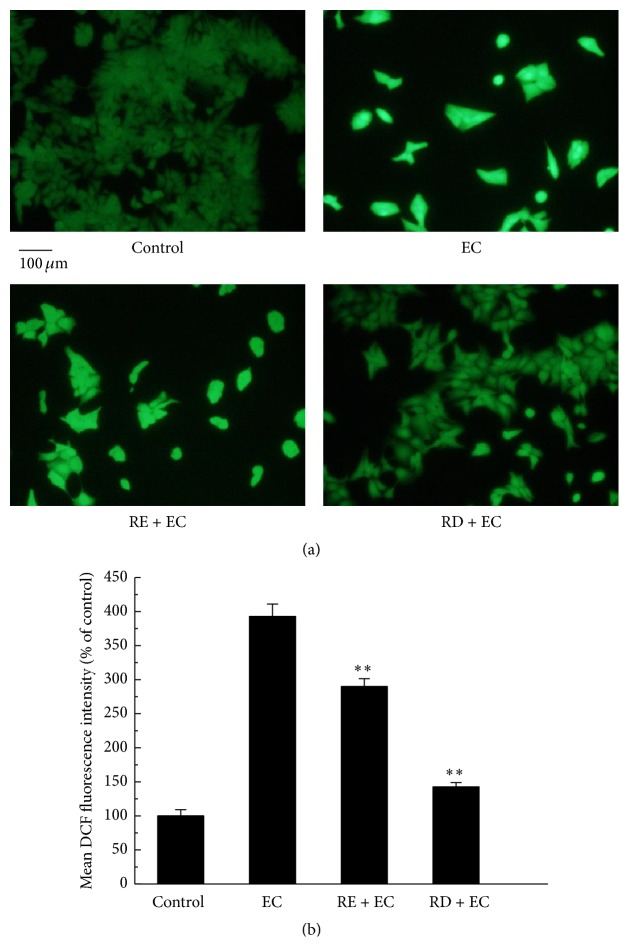
Effect of RD on EC-induced ROS generation in Caco-2 cells. (a) After treatment with 62.5 mM EC in the presence or absence of RE (2 mg/mL) or RD (2 mg/mL) for 24 h, cells were collected and incubated with 10 *μ*M of DCF at 37°C for 30 min; then cells were washed with PBS and evaluated by fluorescence microscope. (b) The quantitative data of panel (a) and results were expressed as mean DCF fluorescence intensity (mean ± standard deviations). ^*∗*^ *p* < 0.05 represents significant difference compared with EC group.

**Figure 3:**
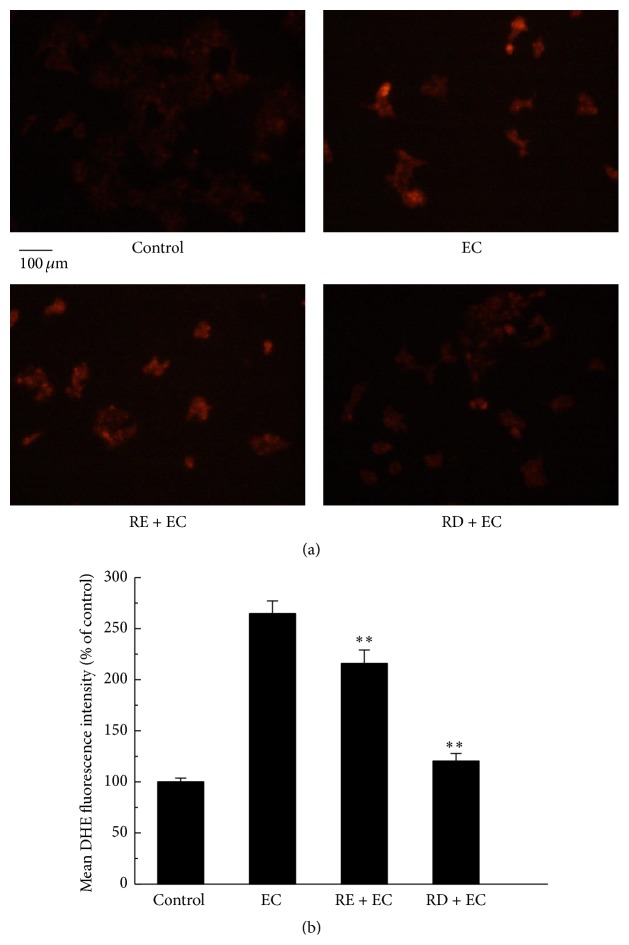
Effect of RD on EC-induced superoxide anion radicals generation in Caco-2 cells. (a) After treatment with 62.5 mM EC in the presence or absence of RE (2 mg/mL) or RD (2 mg/mL) for 24 h, cells were collected and incubated with 10 *μ*M of DHE at 37°C for 30 min; then cells were washed with PBS and evaluated by fluorescence microscope. (b) The quantitative data of panel (a) and results were expressed as mean DHE fluorescence intensity (mean ± standard deviations). ^*∗*^ *p* < 0.05 represents significant difference compared with EC group.

It was reported that EC-induced toxicity was related to the generation of ROS in lung epithelial cells [7]. On the basis of our result that EC could cause cytotoxicity in Caco-2 cell, we next studied whether EC could induce ROS overproduction in Caco-2 cells by incubation with DCFH-DA. The result displayed in Figures 2(a) and 2(b) showed that the DCF fluorescence intensity of EC group was increased to 392.76% compared with control group (its fluorescence intensity was considered as 100%), which indicated that large amount of ROS was accumulated in Caco-2 cells after EC treatment. Subsequently, we explored whether the overproduction of ROS could be scavenged by raspberry digesta (RD). As expected, comparing with that of EC group (392.76%), a sharp decrease of fluorescence intensity was observed in RE group and RD group, with the mean fluorescence intensity declining to 289.83% and 142.40%, respectively. In addition, further study (DHE staining experiment) was employed to examine whether EC could induce the generation of intracellular superoxide anion radicals (O_2_^·−^). Similar results were found and presented in Figures 3(a) and 3(b), in which Caco-2 cells pretreated with RE and RD significantly decreased the DHE fluorescence intensity to 215.88% and 120.41%, respectively, compared with solely EC-treated group (its fluorescence intensity reached 264.68%). The reason contributing to these phenomena may be the release of some bioactive phenolic components after* in vitro* digestion [23], since the structure of phenolic compounds that hydroxyl groups linked to phenolic rings were considered to donate electrons and neutralize reactive oxygen species [24]. As we detected that the total phenolic content was increased from 188.43 mg GAE/100 g to 254.60 mg GAE/100 g and the total flavonoid content increased from 78.30 mg RE/100 g to 103.32 mg RE/100 g after* in vitro* digestion. In conclusion, RD may be more effective than RE in terms of scavenging intracellular ROS and superoxide anion radicals in Caco-2 cells.

### 3.3. RD Improved EC-Induced Abrogation of Intracellular Glutathione (GSH)

**Figure 4:**
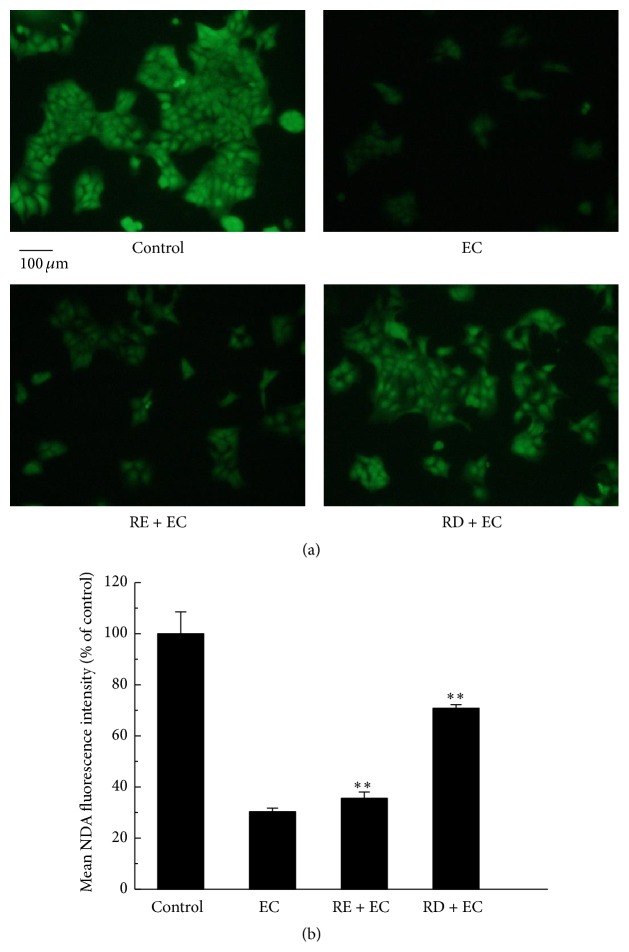
Effect of RD on EC-induced oxidative stress to GSH antioxidant systems in Caco-2 cells. (a) After treatment with 62.5 mM EC in the presence or absence of RE (2 mg/mL) or RD (2 mg/mL) for 24 h. Then cells were collected and incubated with 50 *μ*M NDA at 37°C for 30 min and evaluated by fluorescence microscope. (b) The quantitative data of panel (a) and results were expressed as mean NDA fluorescence intensity (mean ± standard deviations). ^*∗*^ *p* < 0.05 represents significant difference compared with EC group.

Reduced glutathione (GSH) is a key nonenzymatic antioxidant and plays an important role in cellular redox reaction [25]. In several studies, GSH has been identified as an effectively protective agent against oxidative stress [26]. Based on the observation that RD could effectively scavenge intracellular ROS, we thus further investigated the role of RD on intracellular GSH content in the presence or absence of EC. In the present study, cellular GSH level was detected using NDA fluorescence probe. As shown in Figures 4(a) and 4(b), an evident depletion of GSH was observed in EC treatment group, whereas RE and RD pretreatment could effectively attenuate the abrogation of GSH induced by EC. The fluorescence intensities of EC, RE, and RD group were 30.35%, 35.54%, and 70.81%, respectively, compared with that of control group. Together, these results revealed that RD may afford protection against EC-induced glutathione (GSH) depletion.

### 3.4. RD Inhibited EC-Induced Oxidative Damage to Mitochondrial Membrane

**Figure 5:**
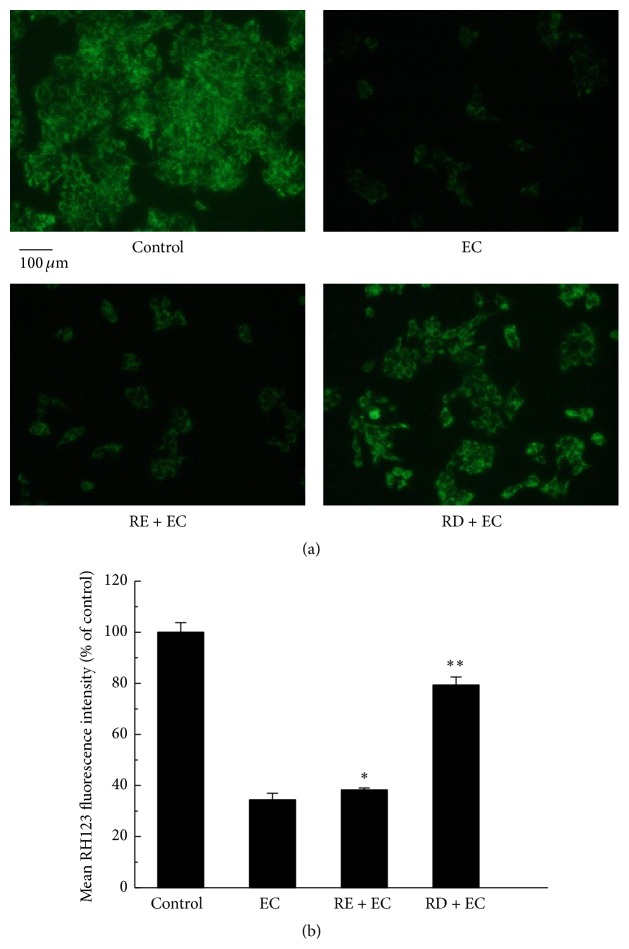
Effect of RD on EC-induced oxidative damage to mitochondrial membrane in Caco-2 cells. (a) After treatment with 62.5 mM EC in the presence or absence of RE (2 mg/mL) or RD (2 mg/mL) for 24 h, Caco-2 cells were incubated with 10 *μ*g/mL RH123 for 30 min and then adopted to fluorescence microscope analysis. (b) The quantitative data of panel (a) and results were expressed as mean RH123 fluorescence intensity (mean ± standard deviations). ^*∗*^ *p* < 0.05 represents significant difference compared with EC group.

Previous reports have revealed that generation of ROS was associated with mitochondrial membrane potential (MMP) collapse [27]. Due to EC-induced accumulation of intracellular ROS, we examined whether EC could cause mitochondrial dysfunction using RH123 fluorescence probe. As expected, the mean fluorescence intensity decreased to 34.49% compared with that of control group (Figures 5(a) and 5(b)), indicating that mitochondrial membrane potential was collapsed in exposure to EC (62.5 mM). We then explored the protective effect of RE or RD on mitochondrial membrane. As shown in Figures 5(a) and 5(b), a potent protective effect of RD was found against MMP collapse triggered by EC, with the mean fluorescence intensity increasing to 79.30% compared with that of EC group, whereas RE treatment only conferred a weak protection (its fluorescence intensity was 38.24%), suggesting that RD may be better than RE in preventing EC-induced mitochondrial membrane impairment.

**Figure 6:**
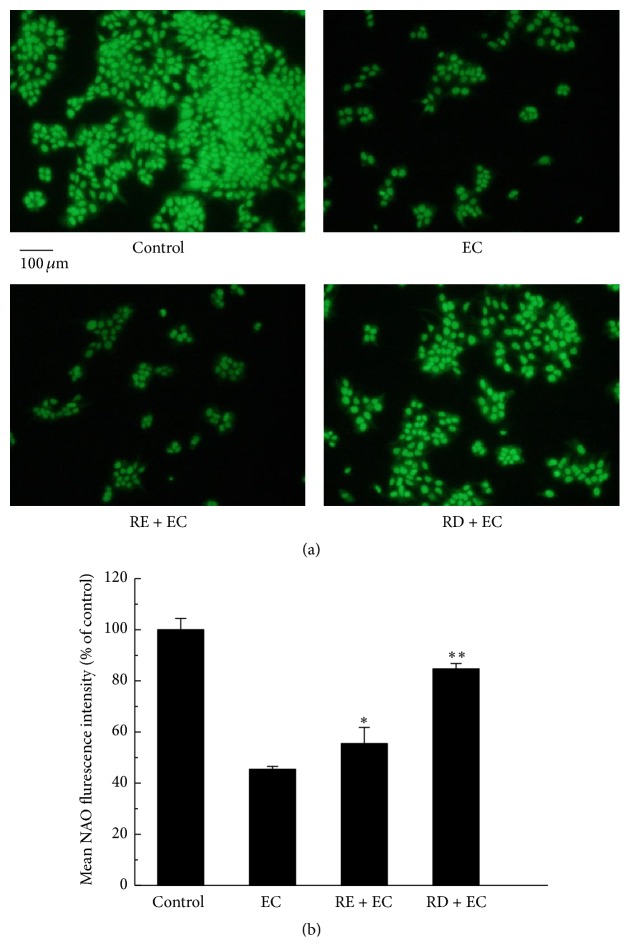
Effect of RD on EC-induced oxidative damage to mitochondrial membrane in Caco-2 cells. (a) After treatment with 62.5 mM EC in the presence or absence of RE (2 mg/mL) or RD (2 mg/mL) for 24 h, Caco-2 cells were incubated with 10 *μ*M NAO for 30 min and subsequently adopted to fluorescence microscope analysis. (b) The quantitative data of panel (a) and results were expressed as mean NAO fluorescence intensity (mean ± standard deviations). ^*∗*^ *p* < 0.05 represents significant difference compared with EC group.

In addition, it is reported that overproduction of ROS can result in mitochondrial membrane lipid peroxidation [28]. Thus, we further studied the effect of RD on suppressing EC-induced lipid peroxidation in Caco-2 cells. NAO, a fluorescence probe, was designed to detect cardiolipin which is a mitochondrial membrane lipid component and would be oxidized in the presence of ROS. As shown in Figures 6(a) and 6(b), without RE or RD pretreatment, massive cardiolipins were oxidized in exposure to EC, with the NAO fluorescence intensity declining to 45.46% compared with that of control group. Nevertheless, after treatment with RE or RD for 2 h, the result turned out to be that their fluorescence intensity increased to 55.47% and 84.70%, respectively, compared with that of EC group (45.46%). On the basis of these results, it can be concluded that RD may afford protection against EC-induced oxidative damage to mitochondrial membrane.

### 3.5. Identification of Phenolic Compounds in RE and RD

**Figure 7:**
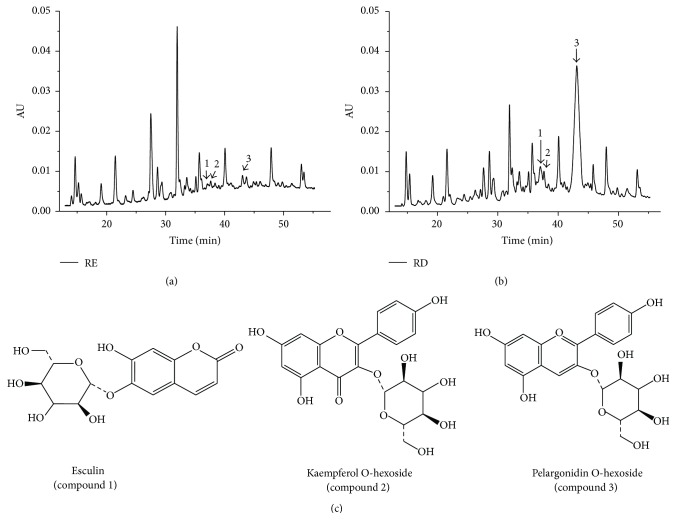
HPLC chromatograms of raspberry extracts produced before and after* simulated* gastrointestinal digestion. (a) Chromatogram of RE. (b) Chromatogram of RD. (c) Chemical structures of identified compounds.

The aforementioned results revealed that EC could result in oxidative stress damage in Caco-2 cells, whereas RD were more effective than RE in attenuating EC-induced oxidative damage. The possible explanation for this phenomenon may be due to structural modification or release of biological active components which were entrapped in food matrix after* in vitro* simulated gastrointestinal digestion [12, 23]. Therefore, HPLC-ESI-MS was employed to analyze the composition of raspberry extracts produced before and after* in vitro* simulated gastrointestinal digestion. HPLC chromatograms displayed the major changes between RE and RD extracts in terms of the content of identified compounds. As we can see from Figures 7(a) and 7(b), the contents of three identified compounds were increased significantly after* in vitro* digestion. According to their MS and MS^2^ data, these compounds were identified as esculin (compound 1) [29], kaempferol O-hexoside (compound 2) [30], and pelargonidin O-hexoside (compound 3) [31], respectively. Berries are rich in phytochemicals, such as anthocyanins, flavonoids, and various phenolic acids, which can provide potent protection against oxidative damage [32, 33]. Kaempferol O-hexoside and pelargonidin O-hexoside are common phytochemicals in berries; they can effectively remove intracellular ROS and increase the total antioxidant activity [8, 34]. Besides, esculin was a novel compound identified in the wild raspberry extract and has been reported to be effective in protecting cells against DNA damage triggered by oxidative stress and scavenging ROS levels [35]. Flavonoids and anthocyanins are mainly located in the vacuoles of cells. Under the simulated gastrointestinal digestion condition, the extreme pH and the enzymatic digestion may fully break down raspberry matrix and vacuoles [36], which leads to the release of these compounds completely. Moreover, some reports considered that the formation of glycoside was increased after digestion probably due to the partial digestion of the dietary fiber present in the matrix [37], which may be beneficial for the formation of anthocyanins including compound 3 (pelargonidin O-hexoside). Overall, we concluded that the better performance of RD in suppressing EC-induced oxidative damage might be partially attributed to these three compounds.

## 4. Conclusion

The present study, for the first time, revealed that EC induced excessive production of intracellular ROS, accelerated GSH depletion and caused mitochondrial dysfunction, which resulted in disturbed cellular redox balance and oxidative damage in Caco-2 cells. Further investigation indicated that* in vitro* simulated gastrointestinal may enhance the ability of wild raspberry extract to suppress EC-induced oxidative stress in Caco-2 cells by scavenging intracellular ROS, preventing GSH depletion, as well as maintaining mitochondrial membrane potential. Moreover, LC-MS results showed that the contents of identified compounds (esculin, kaempferol O-hexoside, and pelargonidin O-hexoside) were significantly increased after* in vitro* digestion, which might be associated with a better biological activity of RD in ameliorating EC-induced oxidative stress. Together, our study indicated that raspberry undergoinggastrointestinal digestion improved its bioactivity, which might have implication for preventing EC-caused health problem.
